# Comparative physiology of canopy tree leaves in evergreen and deciduous forests in lowland Thailand

**DOI:** 10.1038/s41597-023-02468-6

**Published:** 2023-09-08

**Authors:** Atsushi Ishida, Keiko Yamaji, Takashi Nakano, Phanumard Ladpala, Ananya Popradit, Kenichi Yoshimura, Shin-Taro Saiki, Takahisa Maeda, Jin Yoshimura, Kohei Koyama, Sapit Diloksumpun, Dokrak Marod

**Affiliations:** 1 Center for Ecological Research, Kyoto University, Otsu, Shiga, 520-2113 Japan; 2 Graduate School of Life and Environmental Sciences, University of Tsukuba, Tsukuba, Ibaraki, 305-0006 Japan; 3 Yamanashi Mount Fuji Research Institute, Kami-Yoshida, Fuji-Yoshida, Yamanashi, 403-0005 Japan; 4 Department of National Parks, Wildlife and Plant Conservation, Chatuchak, Bangkok, 10900 Thailand; 5 College of Innovation Management, Valaya Alongkorn University under the Royal Patronage, Klongluang, Pathum Thani, 13180 Thailand; 6 Faculty of Agriculture, Yamagata University, Tsuruoka, Yamagata, 997-8555 Japan; 7 Department of Forest Ecology, Forestry and Forest Products Research Institute, Tsukuba, Ibaraki, 305-8687 Japan; 8 National Institute of Advanced Industrial Science and Technology, Tsukuba, Ibaraki, 305-8569 Japan; 9 Institute of Tropical Medicine, Nagasaki University, Sakamoto, Nagasaki, Nagasaki, 852-8523 Japan; 10 Faculty of Science, Tokyo Metropolitan University, Minami-Osawa, Hachioji, Tokyo, 192-0397 Japan; 11 The University Museum, The University of Tokyo, Hongo, Bunkyo, Tokyo, 113-0033 Japan; 12 Asahikawa campus, Hokkaido University of Education, Asahikawa, Hokkaido, 070-8621 Japan; 13 Faculty of Forestry, Kasetsart University, Chatuchak, Bangkok, 10900 Thailand

**Keywords:** Photosynthesis, Forest ecology

## Abstract

The typical seasonally dry forests in Southeast Asia are the mixed deciduous forest (MDF), dry dipterocarp (deciduous) forest (DDF), and dry evergreen forest (DEF). We obtained 21 physiological traits in the top/sunlit leaves of 107, 65 and 51 tree species in MDF, DEF and DDF, respectively. Approximately 70%, 95% and 95% of canopy tree species which consist of MDF, DEF and DDF are sampled, respectively. Light-saturated photosynthetic rates (*A*_sat_) exhibit a positive correlation with foliar nitrogen (N) and phosphorus (P) on leaf mass and area bases across tree species. Decreased leaf mass-based P reduces the positive slope of the mass-based N and *A*_sat_ relationship across species and habitats. The differences in nutrient and water use and leaf habits are well matched to the variation in soil properties among the forest types, highlighting the reliability of this comprehensive database for revealing the mechanism of niche segregation based on edaphic factors.

## Background & Summary

In Thailand, there is a distinct dry season over approximately three months from November to January. There are various forest types in the seasonally dry tropical vegetation of Thailand and the adjacent regions of Southeast Asia. In the tropical dry forests, there are different types from evergreen to drought-deciduous forests, which are geologically separated and dependent on various soil types^1^. The leaf properties and phenology are closely correlated with the habitat soil and climate characteristics with a dry season in Thailand^2–4^. Several studies in tropical rainforests on the island of Borneo have emphasized on the importance of edaphic factors in determining forest structure^5,6^ and function^6–10^. Neotropical dry forests also have high diversity from evergreen, deciduous or semi-deciduous leaf habits, showing different leaf habits^11,12^, and the forest variations in productivity and biomass are strongly dependent on water table depth, chemistry and texture in soil^13,14^. In the seasonally dry forests, which encompass various forest types from evergreen to deciduous in Thailand, it has been hypothesized that the determination of forest composition is strongly dependent on the topography and edaphic factors^2–4^. Nevertheless, compared to the rainforests of Borneo and the Neotropical dry forests, there remains a notable scarcity of comprehensive information on leaf habits in the seasonally dry forests of Southeast Asia, impeding our understanding the establishments of various forest types and the criticality of edaphic factors in this region. Here, we represent a comprehensive data list to clarify the average and variations in leaf physiology of woody plants in the natural forests with different types depending on geology and soil properties in Thailand.

**Fig. 1:**
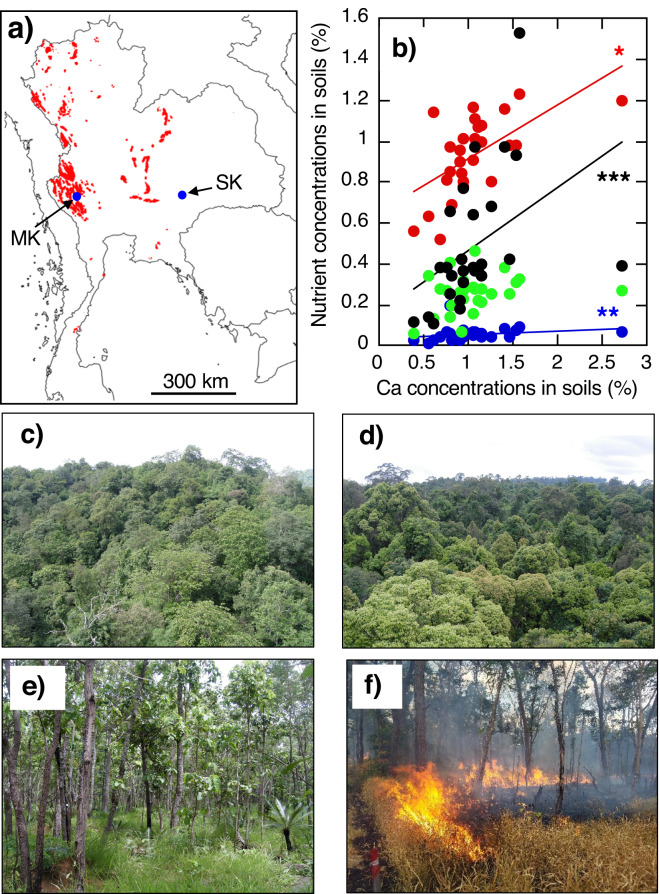
The examined three forest types and study sites. (**a**) The positions of two study sites, Mixed deciduous forest (MDF) at the Mae-Klong Watershed Research Station (MK) and Dry deciduous forest or Dry dipterocarp forest (DDF) and Dry evergreen forest (DEF) at the Sakaerat Environmental Research Station (SK), and the limestone area (red colour) in Thailand. The data source of the limestone area is from the Department of Mineral Resources in Thailand. (**b**) The relationships between Ca and the other nutrients (N: red circle, P: blue circle, K: green circle, Mg: black circle) in the organic (O) horizon of soils over all of Thailand (***P < 0.001, **P < 0.01, n.s.: *P ≥ *0.05). There was no significant correlation between Ca and K. The data source in soil nutrients was Tsutsumi *et al*^17^. (**c**) Photo of MDF in July (rainy season). (**d**) Photo of DEF in July. (**e**) Photo of DDF in July. (**f**) Photo of forest fire in DDF in December (dry season). All photos were taken by A. Ishida (the first author).

The seasonally dry forests in regions below an elevation of 1650 m naturally consist of three main forest types: 1) dry dipterocarp forest (or dry deciduous forest) (DDF), 2) dry evergreen forest (DEF) and 3) mixed deciduous forest (MDF) in Thailand and the adjacent regions^15^. Drought-deciduous trees are predominant in DDF and MDF, while evergreen trees are predominant in DEF. In DDF and MDF, defoliation is found during the dry season. However, the timing of leaf falling and flushing varies greatly depending on the tree species, and even during the dry season, old and new leaves are often mixed together in these deciduous forests^3,16^. On the other hand, MDFs occur primarily in regions with limestone bedrock, predominantly situated in the north-western Thailand, Myanmar and India. The mountain range in north-western Thailand along the border with Myanmar is steep and rugged and cuts into narrow valleys. Few peaks exceed an elevation of 1800 m. The Himalayan orogenic movement formed these steep mountains, and the resulting soil in this area originated from the limestone of the Mesozoic Era (Fig. 1a). In this area, DDFs are scattered on mountain ridges where the soil is extremely shallow.

The Himalayan orogenic movement caused a north-south division through the Chao Phraya River, promoting speciation of trees between the eastern and western parts of Thailand. The north-eastern Thailand consists of the Khorat Plateau (located on the eastern side of the Chao Phraya River), which covers nearly one-third of the country’s area. This area is drained by a tributary of the Mekong River, and the soil originated from sandstone created in the Cenozoic Era. Several hills with gentle slopes from elevations of 650 to 250 m are scattered on this plateau. The tops of hills generally have deep sandy soil extending from the ground surface to the bedrock, while the foots of hills have shallow sandy soil because of soil erosion. These sandy soils have poor nutrient concentrations^1^. In the Khorat Plateau, DEFs usually develop on hill tops with deep sandy soil, whereas DDFs usually develop on foothills with shallow sandy soil. Therefore, the evergreen forests (DEFs) and deciduous forests (DDFs) have separately established within the same hills along the soil thickness gradient in the topography.

Tsutsumi *et al*.^17^ examined the soil nutrients in various forests throughout Thailand, including in MDFs, DDFs and DEFs. Their classical study showed that phosphorus (P), magnesium (Mg) and nitrogen (N) concentrations were positively correlated with calcium (Ca) concentrations in soils (Fig. 1b), probably because of the chemical binding of Ca and P in soil particles, such as Ca(H_2_PO_4_)_2_ and CaHPO_4_. In the study sites, the annual average precipitations in MDF (north-western Thailand) and in DDF and DEF (north-eastern Thailand) are 1240 mm and 1650 mm, respectively; and the annual average air temperatures in MDF and in DDF and DEF are 26.2 °C and 27.5 °C, respectively. The climatological variability encompassing the seasonal patterns may be insufficient enough to create distinct forest formations between deciduous and evergreen forests^2,15^. Therefore, topography and edaphic factors (such as soil nutrients, soil thickness and water table depth) may be more important than climate for the formation of different forest types in these seasonally dry forests in Southeast Asia^2,15^.

A few studies using a limited number of canopy trees have shown that leaf gas exchange, nutrient use, water use and physiological photoprotection during the dry season are different between drought-deciduous trees in MDF and evergreen trees in DEF^2–4^. However, differences of leaf function among these forests have not yet been comprehensively investigated. Thus, the lack of an inventory or a comprehensive data list for tree physiology hinders our understanding of why various forest types are established in Southeast Asia, which has distinct dry seasons, and how forest function varies among forest types. To fill the gaps in knowledge about the forests and their related environments, we have examined the fundamental leaf physiology in the comprehensive canopy trees in MDFs in north-western Thailand, and in DEFs and DDFs in north-eastern Thailand. We obtained 21 physiological traits in the top/sunlit leaves of 107, 65 and 51 tree species in MDF, DEF and DDF, respectively. Approximately 70%, 95% and 95% of canopy tree species which consist of MDF, DEF and DDF are sampled, respectively.

We show that the characteristics of leaf physiology vary among the forest types, and stress the importance of edaphic factors on leaf functional traits, which highlight the reliability of this comprehensive database in revealing the mechanism of niche segregation. Our data indicate that the photosynthetic use efficiency of water and foliar nutrients (not only nitrogen but also phosphorus) is likely to vary with respect to habitat environments, and a decrease in leaf mass-based P reduces the positive slope of the mass-based N and photosynthesis relationship. The fundamental differences in leaf physiology of woody plants are well matched to the differences in soil properties related to nutrient and water resources. The current report provides the first comprehensive dataset of leaf physiology associated with soil properties in the seasonal dry forests in Thailand, and the current dataset will be highly valuable for considering forest function and niche segregation and for meta-analysis globally.

## Methods

### Study sites and tree species list

We selected two study sites: 1) the Mae-Klong Watershed Research Station (14°34′ N, 98°50′ E, 400 m above sea level) in Kanchanaburi Province, approximately 250 km northwest of Bangkok and 2) the Sakaerat Environmental Research Station (14°29′ N, 101°55′ E, 1610 m above sea level) in Nakhon Ratchasima Province, approximately 180 km northeast of Bangkok (Fig. 1a) in Thailand. Figure 1c shows the mixed deciduous forest (MDF) at Mae-Klong, and Fig. 1d shows the dry evergreen forest (DEF) and Fig. 1e show the dry dipterocarp forest or dry deciduous forest at Sakaerat. In the deciduous forests (DDF and MDF), forest fires frequently occur during the late dry season, when leaf litters are accumulated on the forest floor (Fig. 1f).

Drought-deciduous trees are predominant in the top canopies of MDF and DDF, while evergreen trees are predominant in the top canopy of DEF. The basic tree species list for each forest was obtained from Dr. Dokrak Marod (the last author), using a 16 ha plot in each forest. Furthermore, when we found other tree species (not included in the permanent plots) during field measurements, we added those species to the species list. According to the list, the MDF is composed of 157 tree species and 75% of which are drought-deciduous trees; the DDF is composed of 56 tree species and 91% of which are deciduous trees; and the DEF is composed of 69 tree species and 45% of which are deciduous trees. In the DEF, *Hopea ferea* Lanessan (evergreen tree) is predominant.

In MDF at Mae-Klong, the mean annual temperature is 27.5 °C, and the mean annual rainfall is 1650 mm^2^. There is little rainfall between November and February^2–4^. The soils (Kandiustalfs) have a clay to clay loam texture derived from sediment rocks and limestone, are slightly acidic (approximately 5.9 in pH) and are relatively rich in nutrients (approximately 4.1% in total N and 0.16 mg g^−1^ in available P)^15^. The study site is located at a branch of the Khwae Noi River in western Thailand. Although evergreen tree species are found in the valley along the river, many drought-deciduous tree species are generally found on the slopes of mountains. The top-canopy heights are approximately 30 m high above the ground. More detailed information on forest structure and dynamics is given in Marod *et al*.^18^.

In DEF and DDF at Sakaerate, the mean annual temperature is 26.2 °C and the mean annual rainfall is 1240 mm^2^. As with Mae-Klong, there is little rainfall between November and February^2–4^. The study sites are located on a table-mountain hill, ranging in elevation from 650 to 250 m, at a branch of the Mekong River, which dissects the Korat sandstone plateau. The evergreen forest is on a gentle slope facing northeast with a mean inclination of 4° in the upper part of the hill and *Hopea ferrea* Lanessan (Dipterocarpaceae) is the most dominant tree species, especially in the top canopy layer, at this hill site^19^. In contrast, a naturally deciduous forest called DDF is found at the lower part of the hill site with shallow soil (<60 cm). The soils (Tropustults) of this hill are predominantly sandy loams derived from sandstone parent material^19^. They are acidic (approximately 5.3 in pH) with low cation exchange capacities and low nutrient availability (approximately 3.5% in total N and 0.13 mg kg^−1^ in available P)^15^. The top-canopy heights in DEF and DDF are approximately 35 m and 15 m high above the ground, respectively. More detailed information on the landform and soil properties at this study site is given in Murata *et al*.^1^ and Pitman^19^.

### Measurements of leaf gas exchange and chemical analysis

We obtained 21 physiological traits from the top/sunlit leaves of canopy trees in 107, 65 and 51 tree species in MDF, DEF and DDF, respectively (Table 1). In this inventory, approximately 70%, 95% and 95% of canopy tree species that consisted of MDF, DEF and DDF were sampled, respectively.

**Table 1:** The list of plant traits examined and their abbreviations.

Leaf traits	units	Abbreviations
Leaf mass per area	g m^−2^	LMA
Area-based max. net photosynthetic rate (*A*sat)	μmol m^−2^ s^−1^	*A*_a_
Area-based max. water vapour stomatal conductance (*G*max)	mol m^−2^ s^−1^	*G*_a_
Minimum leaf internal CO_2_ concentration	μmol mol^−1^	*C*_i_
Intrinsic water-use efficiency (*A*max/*G*max)	μmol mol^−1^	iWUE
Leaf mass-based nitrogen (N) content	mmol g^−1^	N_m_
Leaf mass-based phosphorus (P) content	μmol g^−1^	P_m_
Leaf mass-based magnisium (Mg) content	μmol g^−1^	Mg_m_
Leaf mass-based calcium (Ca) content	μmol g^−1^	Ca_m_
Leaf mass-based potasium (K) content	μmol g^−1^	K_m_
Leaf area-based N content	mmol m^−2^	N_a_
Leaf area-based P content	mmol m^−2^	P_a_
Leaf area-based Mg content	mmol m^−2^	Mg_a_
Leaf area-based Ca content	mmol m^−2^	Ca_a_
Leaf area-based K content	mmol m^−2^	K_a_
Mass-based Asat	nmol g^−1^ s^−1^	*A*_m_
N-based Asat (Photosynthetic N use efficuency)	μmol molN^−1^ s^−1^	PNUE
P-based Asat (Photosynthetic P use efficuency)	mmol molP^−1^ s^−1^	PPUE
Mg-based Asat (Photosynthetic Mg use efficuency)	mmol molMg^−1^ s^−1^	PMgUE
Leaf carbon/nitrogen ratio		C/N
Leaf δ 13 C ratio	‰	δ13 C

We used the top/sunlit leaves of canopy trees in these natural forests. To determine the maximum leaf gas exchange rates in each tree species, we selected fully expanded, healthy leaves. We usually measured the leaf gas exchange for three to six trees in each tree species during the rainy seasons from 2012 to 2015. If we could not find three adequate trees for a tree species at these sites, we measured the leaf gas exchange for one or two trees. The top/sunlit shoots were collected, using a long carbon pole with a sickle on its head. The cut end of the collected shoot was immediately put into water in a bucket, and then recut in water by pruning scissors. After that, we immediately measured leaf gas exchange. For 3 species out of 107 species in MDF (*Shorea siamensis* Mig., *Xylia xylocarpa* var. *kerrii* (Craib & Hutch.) I.C.Nielsen and *Vitex peduncularis* Wall. Ex Schauer), 3 species out of 65 species in DEF (*Hopea ferrea* Laness., *Shorea henryana* Pierre and *Irvingia malayana* Oliv. ex A.W.Benn.), and 2 species out of 51 species in DDF (*Shorea siamensis* Mig. and *Shorea obtsusa* Wall. ex Blume), we were able to directly access the top canopies using scaffolding towers or crane cars. In these species, we measured leaf gas exchange directly without cutting branches to avoid artificial effects as much as possible. Cutting branches might result in the release of hydraulic limitations and a potential increase in leaf gas exchange rates; the positive effects would be however less than 10% compared to the intact leaves on sunny days^20^.

Measurements of leaf gas exchange were conducted with an open, portable measurement system (LI-6400, LI-COR, Lincoln, NE, USA). The light-saturated net carbon assimilation rate (*A*_sat_) and water vapour stomatal conductance (*G*_max_) were measured for the period from 0900 to 1200 h to avoid midday depression. The measurements were conducted under CO_2_ conditions of 400 μmol mol^−1^ in the inlet gas stream and 2000 μmol m^−2^ s^−1^ with red-blue light-emitting diodes. The relative humidity in the outlet gas stream was maintained at approximately 60%. Leaf temperature was not regulated with the LI-6400. The measured leaf temperatures were 31.7 ± 1.8 °C (Mean ± 1 S.D.), and the measured leaf-to-air vapour pressure differences were 1.61 ± 0.37 kPa (Mean ± 1 S.D.). The concentrations of H_2_O and CO_2_ in the gas stream in the system were determined by infrared spectrophotometry. The intercellular CO_2_ concentration in leaves (*C*_i_) was calculated with the LI-6400. Intrinsic water use efficiency (iWUE) was calculated as follows: iWUE = *A*_sat_/*G*_max_. The maximum leaf gas exchange rates usually vary within a shoot in tropical trees, depending on the leaf age and the degree of self-shading along the twig^21,22^. Thus, we repeatedly measured leaf gas exchange along the shoots collected from one tree and then carefully selected the most vigorous leaf within the shoots.

To determine the leaf mass per area (LMA) and the chemical components within the lamina, we collected leaf discs (approximately 10 discs of 28 mm^2^ in each) from the lamina without thick main veins just after the photosynthetic measurements. When the size of a single leaf was too small, we also used the adjacent leaves along the shoots, because leaves in close proximity exhibit similar characteristics within a shoot^21^. Leaf discs were oven dried (80 °C, 72 h) and weighed to determine the LMA. In this study, the loss of volatile substances and some associated chemicals might be inevitable during the drying process. Using the leaf discs collected from the tree that showed the highest leaf gas exchange rates in each tree species, we measured the chemical concentrations and determined the δ^13^C values (stable carbon isotope ratios). The δ^13^C values were measured with a MAT 525 stable isotope ratio mass spectrometer (Finnigan-MAT, Bremen, Germany) as an index of the long-term averaged leaf internal CO_2_ concentrations^23^. The δ13 C values of dried leaf discs were calculated with reference to the Peedee Belemnite (PDB) standard as follows: δ13 C (‰) = [(*R*_sample_–*R*_PDB_)/*R*_PDB_]1000, where *R*_sample_ and *R*_PDB_ are the ^13^C/^12^C ratio in the samples and the PDB standard (*R*_PDB_ = 0.0112372), respectively.

For chemical analysis, carbon (C) and N concentrations within lamina were measured with an NC-800 N-C analyser (Sumigraph, Sumitomo-Kagaku, Osaka, Japan). The concentrations of P, Ca, Mg and potassium (K) within the lamina were measured, using a solution of leaf discs, as follows. The leaf discs were carefully washed with tap water and then with deionized water prepared by a Millipore filtration system (Merck, Darmstadt, Germany). The cleaned leaf discs were dried at 80 °C for 48 h, and each sample was ground. To quantify nutrient elements within the lamina, the ground materials were weighed and pyrolyzed using concentrated HNO_3_ at 130 °C and then analysed by inductively coupled plasma optical emission spectroscopy (ICP-OES; 720ICP-OES, Agilent Technologies, Santa Clara, CA, USA)^24^. The results of five replications were averaged in each sample. Using the LMA of the leaf discs, we recalculated the leaf area-based values from the leaf mass-based concentrations. In total we obtained 21 plant traits, and the plant trait list and associated abbreviations are shown in Table 1.

## Data Records

All datasets in ecophysiological characteristics in the canopy leaves of each tree species in the three forest types (MDF, DDF and DEF) are available from the Dryad Digital Repository^25^. The number of canopy tree species (including large bamboos reaching the top canopies) in MDF, DEF and DDF at these study sites was 157, 73 and 60, respectively. Out of these trees, the number of tree species examined in the MDF, DEF and DDF was 110, 69 and 57, respectively. Thus, the fill rates in MDF, DEF and DDF reached approximately 70%, 95% and 95%, respectively. To show the potential values in each tree species, the ecophysiological data of a canopy leaf with the maximum leaf gas exchange rate in each tree species are shown.

## Technical Validation

### Leaf function in each forest type

Significant differences were found among the three forest types in 13 plant traits, but not found in the remaining 8 plant traits out of 21 leaf characteristics (Table 2). In particular, MDF (with eutrophic, deep soil) had the lowest iWUE and δ13 C among the three forest types, representing the characteristics of water consumption in the deciduous forest trees with deep limestone soil. The root allocation and the fine-root hydraulic conductance per root surface area can be altered plant hydraulics and iWUE^26^. The lowest δ13 C in MDF also indicated that the long-term averaged *C*_i_ within lamina was certainly high. The photosynthetic enzymes, ribulose-1,5-bisphosphate carboxylase/oxygenase (Rubisco), catalyses two competing reactions involving CO_2_ and O_2_ as substrates. Carboxylation of the substrate ribulose-1,5-bisphosphate leads to photosynthetic carbon assimilation, while the oxygenation reaction competes with carboxylation and reduces photosynthetic productivity. Recently, it has been shown that the affinity of Rubisco to CO_2_ and O_2_ is different among plant species^27–31^. These studies indicate that the interspecific variation in Rubisco affinity is dependent on the variations in the CO_2_/O_2_ ratio at the Rubisco site and in leaf internal air among species. The significant differences in *C*_i_ and δ13 C among the forest types (Table 2) thus suggest that the nutrient metabolism and allocation within lamina varies in relation to Rubisco adaptation to leaf internal CO_2_/O_2_ ratios among the forest types.

**Table 2:** The mean values in each plant trait in each forest type and the significant differences among the forest types.

Site	Plant trait	LMA	*A*_a_	*A*_m_	*G*_a_	*C*_i_	iWUE(*A*/*G*)	N_a_	P_a_	Mg_a_	Ca_a_	K_a_
	unit	g m^−2^	μmol m^−2^ s^−1^	nmol g^−1^ s^−1^	mol m^−2^ s^−1^	μmol mol^−1^	μmol mol^−1^	mmol m^−2^	mmol m^−2^	mmol m^−2^	mmol m^−2^	mmol m^−2^
**MDF**	**AVERAGE**	85.53	14.2	186	0.378	297	41.2	132.1	10.8	11.7	38.3	16.3
	**SD**	24.84	4.0	82	0.156	23	13.5	34.1	2.7	7.9	29.1	7.3
	**N**	87	107	85	107	107	107	84	86	85	86	86
		**b**	**b**	**a**	**a**	**a**	**b**	**b**	**a**	**a**	**a**	**a**
**DDF**	**AVERAGE**	97.00	16.3	179	0.337	272	51.1	151.1	9.7	13.1	30.7	17.6
	**SD**	25.61	3.3	60	0.104	19	12.0	50.2	3.3	7.5	18.7	8.7
	**N**	50	53	50	53	49	53	50	50	50	50	50
		**a**	**a**	**a**	**a**	**b**	**a**	**a**	**b**	**a**	**a**	**a**
**DEF**	**AVERAGE**	102.06	12.9	143	0.263	272	55.3	163.6	9.8	12.7	38.8	18.4
	**SD**	32.11	3.7	78	0.127	30	18.3	39.5	2.2	11.3	26.2	8.4
	**N**	54	65	54	65	65	60	54	54	54	54	54
		**a**	**b**	**b**	**b**	**b**	**a**	**a**	**b**	**a**	**a**	**a**
**One-way ANOVA** ***P*** **values**		**<0.01**	**<0.001**	**<0.01**	**<0.001**	**<0.001**	**<0.001**	**<0.001**	**<0.05**	0.617	0.191	0.415
**Tukey’s test DEF-DDF**		0.612	**<0.001**	**<0.05**	**<0.05**	0.9895	0.276	0.258	0.990	0.968	0.251	0.893
**Tukey’s test MDF-DDF**		<0.05	<0.01	0.845	0.191	**<0.001**	**<0.001**	**<0.05**	**<0.05**	0.627	0.228	0.723
**Tukey’s test MDF-DEF**		**<0.01**	0.067	**<0.01**	**<0.001**	**<0.001**	**<0.001**	**<0.001**	**<0.05**	0.790	0.993	0.400
**Site**	**Plant trait**	**N**_**m**_	**P**_**m**_	**Mg**_**m**_	**Ca**_**m**_	**K**_**m**_	**PNUE**	**PPUE**	**PMgUE**	**C/N**	**δ13 C**	
	**unit**	**mmol g**^**−1**^	**μmol g**^**−1**^	**μmol g**^**−1**^	**μmol g**^**−1**^	**μmol g**^**−1**^	**μmol molN**^**−1**^ **s**^**−1**^	**mmol molP**^**−1**^ **s**^**−1**^	**mmol molMg**^**−1**^ s^**−1**^		**‰**	
**MDF**	**AVERAGE**	1.65	133	135	438	197	113	1.38	1.76	22.2	−30.3	
	**SD**	0.50	39	68	277	84	34	0.38	1.16	7.1	1.5	
	**N**	84	86	85	86	86	84	86	84	84	84	
		**a**	**a**	**a**	**a**	**a**	**a**	**b**	**a**	**a**	**a**	
**DDF**	**AVERAGE**	1.61	104	137	329	191	113	1.84	1.82	23.1	−29.0	
	**SD**	0.46	36	71	205	99	28	0.64	1.29	6.6	1.6	
	**N**	50	50	50	50	50	50	50	50	50	49	
		**a**	**b**	**a**	**b**	**a**	**a**	**a**	**a**	**a**	**b**	
**DEF**	**AVERAGE**	1.74	106	124	381	198	82	1.33	1.67	22.8	−28.9	
	**SD**	0.57	43	85	222	112	29	0.36	1.35	7.7	1.5	
	**N**	54	54	54	54	54	54	54	54	54	54	
		**a**	**b**	**a**	**a,b**	**a**	**b**	**b**	**a**	**a**	**b**	
**One-way ANOVA** ***P*** **values**		0.419	**<0.001**	0.619	**<0.05**	0.891	**<0.001**	**<0.001**	0.816	0.768	**<0.001**	
**Tukey’s test DEF-DDF**		0.398	0.979	0.660	0.529	0.929	**<0.001**	**<0.001**	0.805	0.975	0.900	
**Tukey’s test MDF-DDF**		0.853	**<0.001**	0.991	**<0.05**	0.889	0.998	**<0.001**	0.960	0.769	**<0.001**	
**Tukey’s test MDF-DEF**		0.635	**<0.001**	0.672	0.3719	0.998	**<0.001**	0.898	0.904	0.886	**<0.001**	

The mean and 1 SD values for each plant trait in Mixed deciduous forest (MDF), Dry deciduous forest or Dry dipterocarp forest (DDF) and Dry evergreen forest (DEF) and the statistical results among the forest types (one-way ANOVA). The different letters (a, b, c) show the significant differences among the forest types by Tukey’s test (*P* < 0.05). The N for each plant trait means the number of tree species examined. The abbreviations of plant traits are shown in Table 1.

Using the obtained data, the combinations of differences between the forest types could clarify which plant traits were caused by leaf phenology/longevity (deciduous vs. evergreen) or which plant traits were caused by site-specific soil properties. For example, when the plant traits show the significant differences between the evergreen forest (DEF) and the deciduous forests (MDF and DDF), these traits are caused by variations in leaf phenology/longevity. In contrast, when the plant traits show significant differences between MDF and DDF (deciduous forests), these traits are caused by the site-specific soil properties rather than by the leaf phenology/longevity.

Between the evergreen (DEF) and deciduous forests (MDF and DDF), significant differences were found in three plant traits out of the 21 examined plant traits, showing that variation in these plant traits (evergreen vs. deciduous). The leaf mass-based *A*_sat_ (light-saturated net photosynthetic rate), area-based *G*_max_ (maximum water vapour stomatal conductance), and PNUE (photosynthetic N use efficiency; *A*_sat_/N) were significantly lower in DEF than in DDF and MDF (Table 2). The lowest mass-based *A*_sat_ and PNUE in DEF showed a high N allocation to non-photosynthetic enzymes or other parts (such as the cell wall) within lamina to support their long leaf lifespan^32–34^. Thus, these leaf traits related to photosynthetic N use efficiency and stomatal conductance would be due to the variations in leaf phenology/longevity. On the other hand, between the deciduous forests (MDF and DDF), the significant differences were found in 10 plant traits. LMA (leaf mass per area), area-based *A*_sat_, area-based N, intrinsic water use efficiency (iWUE; *A*_sat_/*G*_max_), PPUE (photosynthetic P use efficiency; *A*_sat_/P) and stable carbon isotope ratio (δ13 C) were significantly lower in MDF (with eutrophic, deep soil) than in DDF (with oligotrophic, shallow soil), while the internal CO_2_ concentration (*C*_i_), area-based P, mass-based P and mass-based Ca were significantly higher in MDF than in DDF (Table 2). Thus, these plant traits related to LMA and photosynthetic P and water use efficiency would be due to the variations in soil properties, but not related to the leaf phenology/longevity.

### Leaf functional change dependent on habitats

The number of tree species commonly observed between two-paired forests was 20 species between DDF and MDF, 17 species between DEF and MDF, and six species between DEF and DDF. Using the dataset of these common species, we examine whether the leaf characteristics are different between the forest types, and investigate the overall trend for each leaf trait using repeated one-way ANOVA. The *P*-values obtained for each plant trait between the two-paired forests are shown in Table 3. The results showed that eight traits out of the 21 examined plant traits were altered significantly according to the habitat environments (*P* < 0.05). In MDF with eutrophic, deep soil, the area-based *G*_max_, mass-based P, area-based P, *C*_i_ and PNUE increased significantly, whereas the iWUE, PPUE and δ13 C decreased significantly even in the same tree species. These data suggest that the photosynthetic use efficiency of water and nutrients (P and N) is likely to vary with respect to habitat environments, indicating these leaf traits are more specific to habitat environments rather than species-specific. In contrast, out of the 21 plant traits of a given tree species, 13 traits (such as LMA, mass-based N, C/N ratio, and area-based and mass-based *A*_sat_) remained unchanged among the three forest types (*P* ≥ 0.05 for all pairs of forests), suggesting that leaf morphology (such as LMA), mass-based N, and photosynthetic rates are more species-specific rather than site- or habitat-specific. Nevertheless, the statistical comparison of all composition species by ANOVA (Table 2) indicates that the averaged LMA among tree species was significantly lower in MDF with eutrophic soil than in DDF with oligotrophic soil, suggesting that the eutrophic soils of MDF selectively favor tree species with lower LMA. It has also been reported that on the island of Borneo, the averaged mass-based P increases and LMA decreases with increasing P availability in soils^6,35^. Thus, the lowest LMA and the highest foliar P in MDF will be well matched to their deciduous phenology with a less conservative nutrient use strategy, especially for P. Bartholomew *et al*.^6^ suggest that the increase in LMA in more nutrient-poor habitats has a compensatory role to partially or fully offset the decrease in mass-based *A*_sat_. However, in the current study, LMA was negatively associated with *A*_sat_ in both leaf mass and area bases (see following Table 4).

**Table 3:** Using the dataset of tree species common between two-paired forests, the overall trends of whether the characteristics of each leaf trait differ between the forest types are examined with repeated one-way ANOVA.

	DDF-MDF	DEF-MDF	DEF-DDF
**LMA**	0.669	0.582	0.725
***A***_**a**_	0.265	0.098	0.368
***G***_**a**_	0.307	**<0.01**	0.894
***C***_**i**_	**<0.01**	**<0.05**	0.719
**iWUE**	**<0.05**	**<0.05**	0.557
**N**_**m**_	0.320	0.064	0.203
**P**_**m**_	**<0.05**	**<0.05**	0.682
**Mg**_**m**_	0.581	0.603	0.279
**Ca**_**m**_	0.744	0.855	0.623
**K**_**m**_	0.476	0.220	0.608
**N**_**a**_	0.304	0.103	0.427
**P**_**a**_	**<0.05**	**<0.05**	**<0.05**
**Mg**_**a**_	0.459	0.439	0.078
**Ca**_**a**_	0.586	0.792	0.436
**K**_**a**_	0.398	0.124	0.733
***A***_**m**_	0.477	0.143	0.714
**PNUE**	**0.2352**	**<0.05**	0.357
**PPUE**	**<0.05**	**<0.001**	0.749
**PMgUE**	0.560	0.723	0.314
**C/N**	0.413	0.215	0.192
**δ13** **C**	**0.1237**	**<0.05**	0.843

The significant differences (*P* < 0.05) in each trait mean that the leaf characteristics are significantly different between the forest types across species. Here, 19, 17 and six common tree species were found between Mixed deciduous forest (MDF) and Dry deciduous forest (DDF), between MDF and Dry evergreen forest (DEF), and between DDF and DEF, respectively. The abbreviations of plant traits are shown in Table 1.

**Table 4:** Summary for multiple regression analyses.

Response traits (Dependent variables)	Explanatory variables in the selected models (Independent variables)	Explanatory variables (Independent variables)	Coefficients (Slopes)	S.E.	Parameter d.f.	Residual d.f.	Student’s *T* values	P values	n
Area-based *A*sat	Site, LMA, Pa, Na, Site:Pa	Intercept	15.27	1.78	9	216	8.59	<0.001***	225
(*A*_a_)		Site(DEF)	−10.64	2.56	9	216	−4.15	<0.001***	225
		Site(MDF)	−8.04	2.15	9	216	−3.74	<0.001***	225
		LMA	−0.03	0.01	9	216	−3.36	<0.001***	225
		Pa	0.11	0.15	9	216	0.77	0.4410	225
		Na	0.02	0.01	9	216	3.08	<0.01**	225
		Site(DEF):Pa	0.72	0.26	9	216	2.84	<0.01**	225
		Site(MDF):Pa	0.56	0.2	9	216	2.83	<0.01**	225
Mass-based *A*sat	Site, LMA, Pm, Nm, Site:Pm	Intercept	220.70	35.51	9	216	6.22	<0.001***	225
(*A*m)		Site(DEF)	−87.42	26.57	9	216	−3.29	<0.01**	225
		Site(MDF)	−122.93	27.36	9	216	−4.49	<0.001***	225
		LMA	−1.02	0.17	9	216	−6.06	<0.001***	225
		Pm	0.11	0.19	9	216	0.61	0.5422	225
		Nm	28.52	9.11	9	216	3.13	<0.01**	225
		Site(DEF):Pm	0.50	0.24	9	216	2.08	<0.05*	225
		Site(MDF):Pm	0.85	0.23	9	216	3.78	<0.001***	225
	Site, LMA, Pm, Nm, Pm:Nm	Intercept	260.22	44.49	8	217	5.85	<0.001***	225
		Site(DEF)	−41.42	9.16	8	217	−4.52	<0.001***	225
		Site(MDF)	−24.45	8.59	8	217	−2.85	<0.01**	225
		LMA	−1.09	0.17	8	217	−6.34	<0.001***	225
		Pm	−0.22	0.28	8	217	−0.79	0.4289	225
		Nm	−13.79	17.51	8	217	−0.79	0.4322	225
		Pm:Nm	0.41	0.13	8	217	3.3	<0.01**	225

The results in the selected regression models of leaf area-based (*A*_a_) or mass-based (*A*_m_) net photosynthetic rates (*A*_sat_) versus leaf mass per area (LMA), leaf area-based or mass-based nitrogen (N) and phosphorous (P), and sites (Site), including their interactions (P:N, Site:P, and Site:N), were shown. According to the AIC values, one model and two models are selected in *A*_a_ and *A*_m_, respectively (see Supplementally Table 1). “Coefficients”, “S.E.”, “d.f.”, and “n” indicate the slope, the standard error, the denominator degree of freedom, and the number of samples in each explanatory variable (independent variable), respectively. Three study sites of DDF (Dry deciduous forest or Dry dipterocarp forest), DEF (Dry evergreen forest), and MDF (Mixed deciduous forest) are used. The values of intercept, Site(DEF) and Site(MDF) correspond to the y-intercept in DDF and its deviations in DEF and MDF, respectively. Due to the significant interactive effects of P variables across different sites in leaf area and mass bases, the slopes of foliar P (phosphorous) correspond to the values in DDF. *P* values indicate the significant levels in the explanatory variables (*** < 0.001, ** < 0.01, * < 0.05).

### Effects of foliar N and P on photosynthesis

**Fig. 2:**
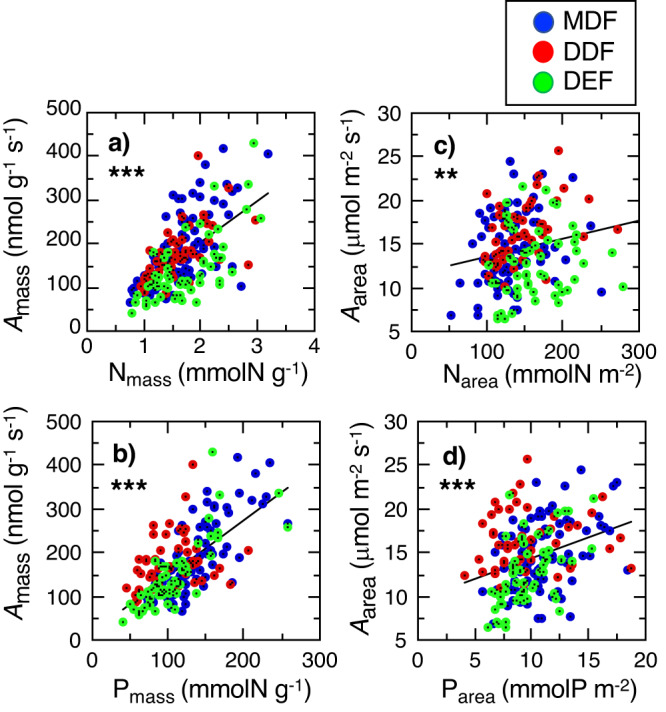
The correlations between light-saturated photosynthetic capacity (*A*_sat_) and foliar phosphorus (P) or nitrogen (N). The relationships (**a,b**) between leaf mass-based *A*_sat_ and leaf mass-based N and P, and (**c,d**) between leaf area-based *A*_sat_ and leaf area-based N and P in Mixed deciduous forest (MDF; blue circles), Dry deciduous forest or Dry dipterocarp forest (DDF; red circles) and Dry evergreen forest (DEF; green circles). The statistical results in Pearson’s correlation in the pooled data are shown in each panel (***P < 0.001, **P < 0.01).

Large interspecific variations in the photosynthetic capacity, *A*_sat_, were found within a forest. In the pooled data (MDF, DDF, DEF), the mass-based *A*_sat_ was positively correlated with the mass-based N and P, and the area-based *A*_sat_ was positively correlated with the area-based N and P (Fig. 2). In leaf area-based and mass-based traits, the effects of LMA, sites, and foliar N and P on the interspecific variation of *A*_sat_ were investigated by a framework of generalized linear models (GLMs), including the interactions of N:P, N:site and P:site (Supplementally Table 1). The regression models selected based on low AIC values led to conclusions regarding the overall effects on the variations of *A*_sat_ (Table 4). Across tree species, an increase in LMA had a significant negative impact on both leaf area-based and mass-based *A*_sat_, while an increase in foliar N and P had a significant positive impact. Furthermore, significant interactions were found between sites and foliar P (Site:P) in both leaf area-based and mass-based *A*_sat_, indicating that the slopes of P-*A*_sat_ relationship across species vary depending on the forest types.

In the mass-based *A*_sat_, a significant interaction were found between foliar P and N (P:N) (Table 4). This fact indicates that an increase in mass-based P leads to an increase in the slope of the N-*A*_sat_ relationship; namely, at a given mass-based N, leaves with lower mass-based P tend to have lower mass-based *A*_sat_ across species and sites. Therefore, this evidence is unlikely to support the conventional hypothesis that there is little or no role in regulating *A*_sat_. It is well known that foliar N is largely invested in photosynthetic enzymes, such as Rubisco, within the lamina^36^, indicating that foliar N is a critical factor for photosynthetic capacity. On the other hand, it has been difficult to clarify the constraint to photosynthetic capacity by foliar P, because foliar P is largely invested in the thylakoid membrane, chlorophyll DNA, sugar phosphate and phospholipids within leaves to regulate and support photosynthesis^37–39^. Nevertheless, it is certain that both foliar N and P are both important elements for enhancing photosynthetic activity in the canopy leaves of the seasonally dry tropical forest trees in Southeast Asia. Because of the decreased sensitivity of photosynthetic capacity to foliar N caused by low P, chronic P deficiency resulting from the poor nutrients and shallow soil in DDF would reduce *A*_sat_. Recent studies on the relationship between the maximum rate of carboxylation (*V*_cmax_) or the maximum rate of electron transport rate (*J*_max_) and foliar P have provided compelling evidence that low P levels have a negative impact on photosynthetic biochemistry in diverse woody species^39–42^. They have also showed that reduced foliar P deceases the positive slope of the N-photosynthetic relationship.

The correlation between two paired plant traits in the pooled data (MDF, DDF, DEF) described the constraint trade-offs in leaf physiology (Table 5). Across many plant species, the correlations between plant traits reveal the trade-off relationships and the physiological diversity in plant evolutionary strategies^6,9,11,43–47^. On the other hand, the plant traits with weak correlations potentially indicate strategic diversities among species^43,45^. In the pooled data (MDF, DDF, DEF), strong positive correlations (*r* > 0.6, *P* < 0.001) were found in 10 pairs: LMA-C/N, *A*_a_-*G*_a_, *A*_a_-*A*_m_, *A*_a_-PNUE, *A*_a_-PPUE, *A*_m_-*G*_a_, *A*_m_-N_m_, *A*_m_-P_m_, Mg_a_-Mg_m_, and Ca_a_-Ca_m_, whereas strong negative correlations (*r* > 0.6, *P* < 0.001) were found in seven pairs: LMA-*N*_m_, LMA-*A*_m_, *G*_a_-iWUE, *C*_i_-iWUE, *N*_m_-C/N, Mg_m_-PMgUE, and Mg_a_-PMgUE (the abbreviations are shown in Table 1). In the pooled data, neither the pairs of P_a_-δ13 C nor P_m_-PPUE were significantly correlated (*P* ≥ 0.05).

**Table 5:** The correlations between plant traits in the pooled data (DEF, DDF and MDF).

	LMA	*A*_a_	*G*_a_	*C*_i_	iWUE	N_m_	P_m_	Mg_m_	Ca_m_	K_m_	N_a_	P_a_	Mg_a_	Ca_a_	K_a_	*A*_m_	PNUE	PPUE	PMgUE	C/N	*δ*13 C
**LMA**	1	n.s.	***(−)	***(−)	***(+)	***(−)	***(−)	n.s.	n.s.	***(−)	***(+)	***(+)	***(+)	***(+)	***(+)	***(−)	***(−)	***(−)	***(−)	***(+)	***(+)
***A***_**a**_	−0.053	1	***(+)	*(−)	*(−)	***(+)	***(+)	n.s.	**(−)	n.s.	**(+)	***(+)	n.s.	**(−)	n.s.	***(+)	***(+)	***(+)	***(+)	**(−)	n.s.
***G***_**a**_	−0.237	0.708	1	***(+)	***(−)	*(+)	***(+)	n.s.	*(−)	*(+)	n.s.	*(+)	n.s.	***(−)	n.s.	***(+)	***(+)	***(+)	***(+)	**(−)	***(−)
***C***_**i**_	−0.266	−0.144	0.524	1	***(−)	n.s.	**(+)	n.s.	n.s.	*(+)	***(−)	n.s.	n.s.	n.s.	n.s.	n.s.	**(+)	n.s.	n.s.	n.s.	***(−)
**iWUE**	0.267	−0.142	−0.774	−0.888	1	n.s.	***(−)	n.s.	n.s.	**(−)	***(+)	n.s.	n.s.	*(+)	n.s.	***(−)	***(−)	n.s.	n.s.	n.s.	***(+)
**N**_**m**_	−0.629	0.239	0.175	−0.088	−0.004	1	***(+)	n.s.	n.s.	***(+)	***(+)	n.s.	***(−)	***(−)	n.s.	***(+)	n.s.	***(+)	***(+)	***(−)	n.s.
**P**_**m**_	−0.653	0.291	0.377	0.202	−0.292	0.520	1	n.s.	*(+)	***(+)	*(−)	***(+)	***(−)	n.s.	n.s.	***(+)	***(+)	n.s.	***(+)	***(−)	**(−)
**Mg**_**m**_	−0.036	−0.110	0.007	0.100	−0.113	−0.024	−0.001	1	***(+)	n.s.	n.s.	n.s.	***(+)	**(+)	n.s.	n.s.	n.s.	n.s.	***(−)	n.s.	n.s.
**Ca**_**m**_	−0.023	−0.220	−0.178	0.050	0.052	−0.008	0.149	0.279	1	n.s.	n.s.	*(+)	***(+)	***(+)	n.s.	n.s.	n.s.	***(−)	***(−)	n.s.	n.s.
**K**_**m**_	−0.408	0.041	0.154	0.186	−0.222	0.345	0.446	0.142	0.115	1	n.s.	n.s.	n.s.	n.s.	***	***	n.s.	n.s.	n.s.	***	**
**N**_**a**_	0.416	0.220	−0.082	−0.440	0.320	0.369	−0.154	−0.069	−0.041	−0.065	1	***(+)	*(+)	*(+)	***(+)	**(−)	***(−)	n.s.	n.s.	***(−)	***(+)
**P**_**a**_	0.271	0.317	0.179	−0.082	−0.034	−0.015	0.494	−0.057	0.168	0.138	0.325	1	n.s.	***(+)	***(+)	n.s.	n.s.	***(−)	n.s.	n.s.	n.s.
**Mg**_**a**_	0.439	−0.114	−0.113	−0.060	0.040	−0.306	−0.295	0.861	0.248	−0.048	0.166	0.100	1	***(+)	***(+)	***(−)	**(−)	*(−)	***(−)	***(+)	n.s.
**Ca**_**a**_	0.393	−0.230	−0.274	−0.118	0.168	−0.265	−0.128	0.232	0.895	−0.067	0.152	0.286	0.412	1	**(+)	***(−)	***(−)	***(−)	***(−)	n.s.	n.s.
**K**_**a**_	0.268	0.011	−0.027	−0.027	−0.018	−0.068	0.001	0.110	0.084	0.742	0.349	0.349	0.253	0.194	1	**(−)	*(−)	***(−)	**(−)	n.s.	n.s.
***A***_**m**_	−0.739	0.676	0.632	0.100	−0.291	0.601	0.667	−0.044	−0.131	0.304	−0.207	0.007	−0.389	−0.435	−0.207	1	***(+)	***(+)	***(+)	***(−)	*(−)
**PNUE**	−0.388	0.645	0.660	0.224	−0.377	−0.074	0.367	−0.023	−0.123	0.121	−0.534	−0.001	−0.223	−0.299	−0.168	0.714	1	***(+)	***(+)	***(+)	(−)
**PPUE**	−0.290	0.608	0.472	−0.058	−0.111	0.242	−0.116	−0.003	−0.323	−0.080	−0.083	−0.493	−0.156	−0.444	−0.295	0.615	0.593	1	***(+)	***(−)	n.s.
**PMgUE**	−0.414	0.493	0.376	−0.009	−0.008	0.381	0.388	−0.779	−0.285	0.071	−0.049	0.047	−0.905	−0.440	−0.212	0.622	0.452	0.380	1	***(−)	n.s.
**C/N**	0.639	−0.193	−0.202	0.005	0.078	−0.927	−0.511	−0.007	−0.103	−0.338	−0.282	0.050	0.283	0.172	0.081	−0.572	0.440	−0.243	−0.343	1	n.s.
**δ13 C**	0.290	0.051	−0.262	−0.486	0.432	0.015	−0.194	−0.061	0.005	−0.193	0.373	0.119	0.010	0.135	0.021	−0.177	−0.257	−0.061	−0.064	0.031	1

Spearman’s rank correlation was conducted for each pair of plant traits. The correlation coefficient (*r*) is given in the lower left section. In the upper right section, (+) shows a positive correlation and (−) shows a negative correlation for each pair (***P < 0.001, **P < 0.01, *P < 0.05, n.s.: *P* ≥ 0.05). The abbreviations of plant traits are shown in Table 1.

The correlations between two paired plant traits in each forest type are shown in Tables 6–8 in DEF, DDF and MDF, respectively. Comparing the plant trait correlations of each forest with those of the pooled data in Table 5, a significant positive correlation was found in the pairs of P_a_-δ13 C in DEF and MDF, while a significant negative correlation was found in the pair of P_m_-PPUE in DDF. Namely, in DEF and MDF, an increase in area-based P suggests an enhancement of long-term water use efficiency (i.e., active carbon fixation and/or water conservation) across species. On the other hand, in DDF with oligotrophic shallow soil, a decrease in mass-based P suggests an increase in the allocation of P to organs associated with photosynthesis within lamina across species. These differences indicate that each forest type could have specific leaf characteristics within a forest.

**Table 6:** The correlations between plant traits in Dry evergreen forest (DEF).

	LMA	*A*_a_	*G*_a_	*C*_i_	iWUE	N_m_	P_m_	Mg_m_	Ca_m_	K_m_	N_a_	P_a_	Mg_a_	Ca_a_	K_a_	*A*_m_	PNUE	PPUE	PMgUE	C/N	δ13 C
**LMA**	1	n.s.	n.s.	*(−)	*(+)	***(−)	***(−)	n.s.	n.s.	***(−)	*(+)	n.s.	**(+)	**(+)	n.s.	***(−)	*(−)	*(−)	**(−)	***(+)	n.s.
***A***_**a**_	−0.083	1	***(+)	n.s.	n.s.	n.s.	**(+)	n.s.	n.s.	n.s.	n.s.	***(+)	n.s.	n.s.	n.s.	***(+)	***(+)	***(+)	***(+)	n.s.	n.s.
***G***_**a**_	−0.258	0.726	1	***(+)	***(−)	n.s.	***(+)	n.s.	n.s.	n.s.	n.s.	*(+)	n.s.	n.s.	n.s.	***(+)	***(+)	***(+)	**(+)	n.s.	n.s.
***C***_**i**_	−0.258	0.016	0.642	1	***(−)	n.s.	n.s.	n.s.	n.s.	*(+)	**(−)	*(−)	n.s.	n.s.	n.s.	n.s.	*(+)	n.s.	n.s.	n.s.	***(−)
**iWUE**	0.301	−0.228	−0.799	−0.923	1	n.s.	*(−)	n.s.	n.s.	*(−)	**(+)	n.s.	n.s.	n.s.	n.s.	*(−)	**(−)	*(−)	n.s.	n.s.	*(+)
**N**_**m**_	−0.748	0.133	0.205	0.043	−0.045	1	***(+)	n.s.	n.s.	***(+)	*(+)	n.s.	*(−)	*(−)	n.s.	***(+)	n.s.	n.s.	**(+)	***(−)	n.s.
**P**_**m**_	−0.771	0.417	0.463	0.176	−0.280	0.757	1	n.s.	n.s.	***(+)	n.s.	***(+)	*(−)	*(−)	n.s.	***(+)	**(+)	n.s.	**(+)	***(−)	n.s.
**Mg**_**m**_	−0.055	−0.133	−0.036	0.067	−0.021	0.073	0.107	1	***(+)	n.s.	n.s.	n.s.	***(+)	**(+)	n.s.	n.s.	n.s.	n.s.	***(−)	n.s.	n.s.
**Ca**_**m**_	−0.004	−0.189	−0.145	0.051	0.044	0.008	−0.006	0.485	1	n.s.	n.s.	n.s.	***(+)	***(+)	n.s.	n.s.	n.s.	n.s.	***(−)	n.s.	n.s.
**K**_**m**_	−0.473	−0.046	0.208	0.275	−0.310	0.519	0.465	0.124	−0.076	1	n.s.	n.s.	n.s.	*(−)	***(+)	*(+)	n.s.	n.s.	n.s.	***(−)	n.s.
**N**_**a**_	0.316	0.178	−0.103	−0.422	0.381	0.343	−0.017	−0.033	0.007	0.005	1	**(+)	n.s.	n.s.	n.s.	n.s.	***(−)	n.s.	n.s.	*(−)	*(+)
**P**_**a**_	0.248	0.544	0.271	−0.277	0.096	0.084	0.355	−0.064	−0.007	0.003	0.355	1	n.s.	n.s.	n.s.	n.s.	n.s.	n.s.	n.s.	n.s.	**(+)
**Mg**_**a**_	0.430	−0.166	−0.159	−0.084	0.119	−0.284	−0.275	0.858	0.463	−0.130	0.140	0.074	1	***(+)	n.s.	**(−)	n.s.	n.s.	***(−)	n.s.	n.s.
**Ca**_**a**_	0.417	−0.221	−0.252	−0.102	0.176	−0.313	−0.323	0.388	0.895	−0.278	0.146	0.119	0.577	1	n.s.	**(−)	*(−)	*(−)	***(−)	*(+)	n.s.
**K**_**a**_	0.152	−0.150	0.007	0.083	−0.120	0.050	−0.043	0.096	−0.109	0.772	0.267	0.139	0.144	−0.052	1	n.s.	*(−)	n.s.	n.s.	n.s.	n.s.
***A***_**m**_	−0.733	0.698	0.648	0.193	−0.317	0.669	0.823	0.009	−0.090	0.276	−0.091	0.187	−0.355	−0.407	−0.230	1	***(+)	***(+)	***(+)	***(−)	n.s.
**PNUE**	−0.288	0.766	0.733	0.272	−0.428	−0.009	0.393	−0.058	−0.182	−0.038	−0.457	0.154	−0.214	−0.304	−0.277	0.698	1	***(+)	**(+)	n.s.	n.s.
**PPUE**	−0.274	0.683	0.603	0.200	−0.286	0.180	0.185	−0.065	−0.223	−0.066	−0.171	−0.165	−0.207	−0.347	−0.050	0.647	0.739	1	**(+)	n.s.	n.s.
**PMgUE**	−0.412	0.499	0.418	0.100	−0.196	0.372	0.403	−0.779	−0.444	0.146	0.008	0.151	−0.917	−0.561	−0.139	0.570	0.427	0.425	1	**(−)	n.s.
**C/N**	0.753	−0.226	−0.268	−0.113	0.118	−0.983	−0.775	−0.116	−0.059	−0.517	−0.306	−0.093	0.251	0.271	−0.039	−0.690	−0.035	−0.200	−0.357	1	n.s.
***δ*13** **C**	0.245	0.110	−0.176	−0.450	0.338	−0.110	−0.049	−0.046	−0.020	−0.146	0.318	0.356	0.063	0.110	−0.018	−0.045	−0.101	−0.061	0.010	0.108	1

Spearman’s rank correlation was conducted for each pair of plant traits. The correlation coefficient (*r*) is given in the lower left section. In the upper right section, (+) shows a positive correlation and (−) shows a negative correlation for each pair (***P < 0.001, **P < 0.01, *P < 0.05, n.s.: *P* ≥ 0.05). The abbreviations of plant traits are shown in Table 1.

**Table 7:** The correlations between plant traits in Dry deciduous forest or Dry dipterocarp forest (DDF).

	LMA	*A*_a_	*G*_a_	*C*_i_	iWUE	N_m_	P_m_	Mg_m_	Ca_m_	K_m_	N_a_	P_a_	Mg_a_	Ca_a_	K_a_	*A*_m_	PNUE	PPUE	PMgUE	C/N	*δ*13 C
LMA	1	n.s.	n.s.	n.s.	n.s.	***(−)	**(−)	n.s.	n.s.	**(−)	***(+)	n.s.	*(+)	n.s.	n.s.	***(−)	**(−)	n.s.	n.s.	**(+)	n.s.
*A*_a_	0.164	1	***(+)	n.s.	n.s.	*(+)	n.s.	***(−)	n.s.	n.s.	***(+)	n.s.	**(−)	n.s.	n.s.	***(+)	*(+)	**(+)	***(+)	n.s.	n.s.
*G*_a_	−0.019	0.694	1	***(+)	***(−)	n.s.	n.s.	n.s.	**(−)	n.s.	n.s.	n.s.	n.s.	*(−)	n.s.	**(+)	*(+)	**(+)	*(+)	n.s.	n.s.
*C*_i_	−0.253	−0.162	0.551	1	***(−)	n.s.	n.s.	n.s.	n.s.	n.s.	*(−)	n.s.	n.s.	n.s.	n.s.	n.s.	n.s.	n.s.	n.s.	n.s.	n.s.
iWUE	0.167	−0.162	−0.793	−0.925	1	n.s.	n.s.	n.s.	n.s.	n.s.	n.s.	n.s.	n.s.	*(+)	n.s.	n.s.	n.s.	n.s.	n.s.	n.s.	n.s.
N_m_	−0.528	0.311	0.274	−0.056	−0.093	1	*(+)	n.s.	n.s.	n.s.	**(+)	n.s.	*(−)	n.s.	n.s.	***(+)	n.s.	*(+)	**(+)	***(−)	n.s.
P_m_	−0.422	0.027	0.036	0.052	−0.064	0.284	1	n.s.	***(+)	***(+)	n.s.	***(+)	n.s.	*(+)	n.s.	**(+)	n.s.	***(−)	n.s.	n.s.	n.s.
Mg_m_	−0.105	−0.507	−0.196	0.239	−0.142	−0.096	−0.053	1	*(+)	n.s.	n.s.	n.s.	***(+)	*(+)	n.s.	n.s.	n.s.	n.s.	***(−)	n.s.	n.s.
Ca_m_	−0.199	−0.218	−0.329	−0.205	0.236	0.061	0.463	0.313	1	*(+)	n.s.	**(+)	n.s.	***(+)	n.s.	n.s.	n.s.	***(−)	n.s.	n.s.	n.s.
K_m_	−0.455	−0.099	−0.026	0.051	−0.085	0.257	0.493	0.215	0.304	1	n.s.	*(+)	n.s.	n.s.	***(+)	*(+)	n.s.	n.s.	n.s.	n.s.	n.s.
N_a_	0.488	0.516	0.271	−0.292	0.067	0.403	−0.098	−0.202	−0.144	−0.165	1	n.s.	n.s.	n.s.	n.s.	n.s.	***(−)	n.s.	n.s.	**(−)	n.s.
P_a_	0.230	0.093	−0.044	−0.144	0.095	−0.061	0.725	−0.111	0.384	0.290	0.265	1	n.s.	***(+)	**(+)	n.s.	n.s.	***(−)	n.s.	n.s.	n.s.
Mg_a_	0.342	−0.390	−0.181	0.150	−0.074	−0.326	−0.210	0.868	0.211	0.059	0.058	0.068	1	**(+)	n.s.	***(−)	**(−)	*(−)	***(−)	*(+)	n.s.
Ca_a_	0.184	−0.160	−0.333	−0.263	0.281	−0.113	0.313	0.301	0.900	0.174	0.084	0.511	0.390	1	n.s.	*(−)	n.s.	***(−)	**(−)	n.s.	n.s.
K_a_	0.048	−0.010	−0.049	−0.093	0.013	−0.002	0.276	0.149	0.191	0.844	0.128	0.445	0.255	0.276	1	n.s.	n.s.	**(−)	n.s.	n.s.	n.s.
*A*_m_	−0.755	0.468	0.412	0.019	−0.175	0.653	0.370	−0.274	0.027	0.291	−0.112	−0.178	−0.755	−0.279	−0.108	1	***(+)	***(+)	***(+)	***(−)	n.s.
PNUE	−0.342	0.294	0.313	0.144	−0.194	−0.238	0.083	−0.192	−0.051	0.082	−0.590	−0.211	−0.357	−0.222	−0.130	0.517	1	**(+)	**(+)	*(+)	n.s.
PPUE	−0.220	0.418	0.422	0.058	−0.195	0.301	−0.585	−0.159	−0.455	−0.252	0.016	−0.826	−0.296	−0.577	−0.388	0.472	0.344	1	**(+)	**(−)	n.s.
PMgUE	−0.270	0.592	0.349	−0.164	0.009	0.385	0.232	−0.884	−0.200	−0.053	0.010	0.001	−0.960	−0.346	−0.202	0.644	0.386	0.341	1	**(−)	n.s.
C/N	0.419	−0.238	−0.167	0.114	0.021	−0.850	−0.127	0.120	−0.151	−0.216	−0.363	0.132	0.029	0.002	−0.024	−0.513	0.283	−0.337	−0.338	1	n.s.
*δ*13 C	0.203	0.027	−0.085	−0.153	0.192	−0.054	−0.107	−0.004	0.048	−0.267	0.131	0.101	0.081	0.119	−0.163	−0.187	−0.144	−0.093	−0.091	0.083	1

Spearman’s rank correlation was conducted for each pair of plant traits. The correlation coefficient (*r*) is given in the lower left section. In the upper right section, (+) shows a positive correlation and (−) shows a negative correlation for each pair (***P < 0.001, **P < 0.01, *P < 0.05, n.s.: *P* ≥ 0.05). The abbreviations of plant traits are shown in Table 1.

**Table 8:** The correlations between plant traits in Mixed deciduous forest (MDF).

	LMA	*A*_a_	*G*_a_	*C*_i_	iWUE	N_m_	P_m_	Mg_m_	Ca_m_	K_m_	N_a_	P_a_	Mg_a_	Ca_a_	K_a_	*A*_m_	PNUE	PPUE	PMgUE	C/N	13 C
LMA	1	n.s.	n.s.	n.s.	n.s.	***(−)	***(−)	n.s.	n.s.	**(−)	***(+)	***(+)	***(+)	***(+)	***(+)	***(−)	***(−)	***(−)	***(−)	***(+)	*(+)
*A*_a_	######	1	***(+)	**(−)	n.s.	**(+)	***(+)	n.s.	*(−)	n.s.	**(+)	***(+)	n.s.	*(−)	n.s.	***(+)	***(+)	***(+)	***(+)	*(−)	n.s.
*G*_a_	######	0.733	1	***(+)	***(−)	n.s.	***(+)	n.s.	**(−)	n.s.	n.s.	*(+)	n.s.	*(−)	n.s.	***(+)	***(+)	***(+)	**(+)	n.s.	*(−)
*C*_i_	######	######	0.315	1	***(−)	*(−)	n.s.	n.s.	n.s.	n.s.	***(−)	*(−)	n.s.	n.s.	n.s.	n.s.	n.s.	n.s.	n.s.	n.s.	***(−)
iWUE	0.118	######	######	######	1	n.s.	n.s.	n.s.	n.s.	n.s.	n.s.	n.s.	n.s.	n.s.	n.s.	n.s.	*(−)	n.s.	n.s.	n.s.	***(+)
N_m_	######	0.341	0.193	######	0.073	1	***(+)	n.s.	n.s.	**(+)	***(+)	n.s.	**(−)	**(−)	n.s.	***(+)	n.s.	**(+)	***(+)	***(+)	n.s.
P_m_	######	0.473	0.401	######	######	0.647	1	n.s.	n.s.	***(+)	n.s.	***(+)	***(−)	**(−)	n.s.	***(+)	***(+)	n.s.	***(+)	***(−)	n.s.
Mg_m_	0.055	0.040	0.081	0.010	######	######	######	1	n.s.	n.s.	n.s.	n.s.	***(+)	n.s.	n.s.	n.s.	n.s.	n.s.	***(−)	n.s.	n.s.
Ca_m_	0.109	######	######	######	0.068	######	######	0.162	1	n.s.	n.s.	n.s.	n.s.	***(+)	n.s.	*(−)	n.s.	**(−)	*(−)	n.s.	n.s.
K_m_	######	0.200	0.188	0.062	######	0.292	0.419	0.119	0.152	1	n.s.	n.s.	n.s.	n.s.	n.s.	**(+)	n.s.	n.s.	n.s.	**(−)	n.s.
N_a_	0.354	0.286	0.076	######	0.198	0.388	0.037	0.107	0.065	0.015	1	***(+)	**(+)	*(+)	*(+)	n.s.	***(−)	n.s.	n.s.	*(−)	**(+)
P_a_	0.457	0.422	0.218	######	0.023	######	0.371	######	0.080	0.169	0.473	1	n.s.	*(+)	***(+)	n.s.	n.s.	***(−)	n.s.	n.s.	**(+)
Mg_a_	0.528	######	######	######	######	######	######	0.853	0.192	######	0.308	0.208	1	***(+)	***(+)	***(−)	*(−)	n.s.	***(−)	**(+)	n.s.
Ca_a_	0.487	######	######	######	0.123	######	######	0.140	0.902	######	0.221	0.273	0.373	1	**(+)	***(−)	**(−)	***(−)	***(−)	n.s.	n.s.
K_a_	0.458	0.113	0.039	######	######	######	######	0.137	0.174	0.687	0.245	0.491	0.336	0.322	1	*(−)	n.s.	**(−)	*(−)	*(+)	n.s.
*A*_m_	######	0.718	0.586	######	######	0.666	0.751	######	######	0.311	######	######	######	######	######	1	***(+)	***(+)	***(+)	***(−)	n.s.
PNUE	######	0.617	0.606	0.032	######	0.016	0.367	######	######	0.173	######	######	######	######	######	0.712	1	***(+)	***(+)	n.s.	n.s.
PPUE	######	0.602	0.501	######	######	0.352	0.163	0.094	######	0.051	######	######	######	######	######	0.751	0.728	1	***(+)	**(−)	n.s.
PMgUE	######	0.440	0.315	######	0.011	0.431	0.550	######	######	0.121	######	######	######	######	######	0.652	0.498	0.413	1	***(−)	n.s.
C/N	0.682	######	######	0.158	0.035	######	######	######	######	######	######	0.111	0.301	0.187	0.231	######	−0.0251	######	−0.377	1	n.s.
13 C	0.223	0.104	######	######	0.417	0.097	0.010	######	0.093	######	0.356	0.289	0.120	0.171	0.106	######	−0.199	######	−0.059	######	1

Spearman’s rank correlation was conducted for each pair of plant traits. The correlation coefficient (*r*) is given in the lower left section. In the upper right section, (+) shows a positive correlation and (−) shows a negative correlation for each pair (***P < 0.001, **P < 0.01, *P < 0.05, n.s.: *P* ≥ 0.05). The abbreviations of plant traits are shown in Table 1.

### Niche segregation of forests

**Fig. 3:**
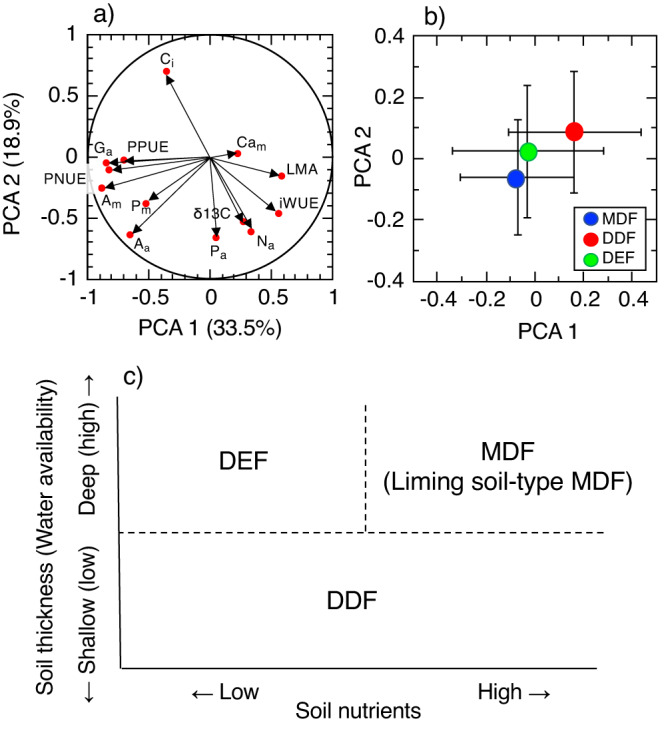
The statistical variations among three forest types. Principal component analysis for **(a**) 13 plant traits whose significant differences among the forest types are recognized in Table 2 and (**b**) the mean values of ±1 S.E. in the tree species of MDF (blue circle), DEF (green circle) and DDF (red circle). The plant trait codes in (**a**) are shown in Table 1. (**c**) Schematic diagram of the distribution of three different forest types, DDF (Dry dipterocarp forest or Dry deciduous forest), MDF (Mixed deciduous forest) and DEF (Dry evergreen forest), along the axes of soil thickness (soil water) and soil nutrients availability as the forest establishment factors.

The principal component analysis (PCA) shows different leaf function among the forest types, and reveals that the nutrient use within the lamina (especially in P and N) and the leaf area-based performance drive the functional diversity among the three forest types (Fig. 3a,b). The first three axes accounted for 66.5% of the total variation; specifically axes 1, 2 and 3 explained 33.5%, 18.9% and 14.1% of the total variations, respectively. Axis 1 was associated with leaf thickness and nutrient use within the lamina, and it was positively correlated with LMA and negatively correlated with the mass-based *A*_sat_, area-based *G*_max_, PNUE and PPUE. Axis 2 was associated with leaf area-based performance, and it was positively correlated with *C*_i_ and negatively correlated with the δ13 C, area-based *A*_sat_, area-based P and area-based N. Axis 3 was positively correlated with *C*_i_ and area-based P and negatively correlated with iWUE (data not shown). These results indicate that the leaf function varies among the forest types, especially in LMA, leaf area-based gas exchange rates and nutrient use of leaves (especially in P and N).

Because there is a distinct dry season lasting several months, the available water in soil should be the most major limiting factor on tree survival and performance and forest function in Thailand^3,48^. However, there are various forest types from evergreen to drought-deciduous forests in Southeast Asia. The combinations of soil thickness and available nutrients would be the essential environmental factors determining the resulting forest types (Fig. 3c). Although DDF is the deciduous and DEF is the evergreen forests at Sakaerat Environmental Station (sandy soil site), they are closer together than the liming soil-type MDF in the PCA (Fig. 3b) illuminating the importance of edaphic factors for establishing different forest types. The deciduous trees in MDF (limestone area) showed a less conservative use of resources, such as nutrients and water. However, MDFs exist partially in sandy soil sites with poor nutrients in the Khorat Plateau, north-eastern Thailand^49^. At Sakaerat Environmental Station (sandy soil site) the top-canopy heights of trees gradually increase with soil thickness from DDF to DEF sites, forming ecotone forests. Leaf and forest function of MDF in sandy soil sites could be different from the MDF examined in the current study (limestone site), suggesting that MDFs include two forest types of ecotone-type MDF (sandy soil-type MDF) and liming soil-type MDF. More research is needed to compare the tree physiology and forest function of MDFs in Southeast Asia.

Our data provide evidence supporting the pivotal role of topography and edaphic factors (soil nutrients and water) in determining the habitat associations of diverse forest types and corresponding leaf functional traits within the seasonally dry tropical forests. The different patterns of water-use and nutrient-use (especially in N and P) of trees among the forest types emerge as notable mechanisms governing the assembly of forest communities, highlighting the fundamental influence of two main soil types originating from limestone in MDF and sandstone in DEF. These differences substantially arise from the soil types attributed to the predominance of ancient bedrock between regions. Furthermore, our data indicate that a decrease in mass-based P reduces the positive slope of mass-based N-*A*_sat_ relationships across species and habitats. Ellsworth *et al*.^38^ demonstrate that incorporating foliar P constraints in a global model significantly reduced gross photosynthesis across the tropics and subtropics by 36%, compared to conventional N constraints and unrestricted P conditions. The canopy leaves of MDF, which have the highest mass-base P and the lowest LMA among the forest types (Table 2), reflect a less conservative resource use of trees and contribute to a rapid nutrient recycling within the forest ecosystems with eutrophic soil. Therefore, the diversity of nutrient use and leaf longevity of trees among forest types will directly influence forest function and strengthen the connection between the soil and the composing trees.

As a limitation of the current dataset, photosynthetically biochemical parameters, such as *V*_cmax_ and *J*_max_, have not been investigated, and the examined *A*_sat_ is dependent not only on *V*_cmax_ but also on stomatal limitation and leaf internal CO_2_ diffusion resistance. Moreover, in tropical trees, their leaf function is influenced by tree height variations^6,50^ even within a tree species^51^, as well as by the developmental stage (ontogeny) of individual trees^52^. In DDF, resistance against forest fire (such as thick bark) should be an important factor for tree survival, because thick bark helps prevent cambium temperatures from increasing to lethal levels during forest fires^53–55^. However, these factors are not yet incorporated into the current dataset. Some climate models predict that in the 21st century, precipitation will increase during the rainy season and will decrease during the dry season in Thailand under global climate change^56^. Prolonged drought causes fatal damage to adult trees by carbon starvation^57–59^ or hydraulic failure^60^ in tropical forests. Recently, anthropogenic impacts have been increasing globally in tropical dry forests, requiring a high priority for conservation^61^. The accelerated development of agricultural land progresses forest fragmentation, resulting in vegetation changes particularly in the peripheral areas of the forest^49^. The amounts of sulfur oxides deposited from the atmosphere to plants and soil has been increasing in Thailand, resulting in changes in the chemical properties of forests^62^. Such enhanced anthropogenic influences and climate change may significantly impact the forest function and their habitat associations in future. The current dataset will not only be relevant to the response of forests to environmental changes but also provide insights into the restoration of degraded forests.

### Supplementary information

Supplementary Table 1

## Data Availability

All statistical analyses were conducted with the software packages of “R” Ver. 4.1.1 (R Development Core Team, 2019)^63^ and “EZR”^64^. All statistical significances were recognized by *P* < 0.05 (****P* < 0.001, ***P* < 0.01, **P* < 0.05, n.s.: *P* ≥ 0.05).
