# Supplementary material for: Comparative physiology of canopy tree leaves in evergreen and deciduous forests in lowland Thailand
Source: Sci Data. 2023 Sep 8;10:601. doi: 10.1038/s41597-023-02468-6 (PMC10491629; doi:10.1038/s41597-023-02468-6)
Supplement: Supplementary file 1 — Supplementary Table 1 [file 41597_2023_2468_MOESM1_ESM.pdf]

**Supplementary Table 1.** The AIC values for selecting the best fit models within the multiple regression analyses. The results in each generalized linear model of leaf area-based ($A_a$) or mass-based ($A_m$) net photosynthetic rates ($A_{sat}$) versus leaf mass per area (LMA), leaf area-based or mass-based nitrogen (N) and phosphorous (P), and sites (Site), including their interactions (P:N, Site:P, and Site:N), were shown. "d.f." and "n" indicate the denominator degrees of freedom and the number of samples, respectively. According to the AIC values, blue-coloured regressions in $A_a$ and $A_m$ (dependent variables) are selected, respectively.

| Response traits (Dependent variables) | Explanatory variables (Independent variables) | Parameter d.f. | Residual d.f. | n | AIC |
|---|---|---|---|---|---|
| Area-based *A*sat | Site | 3 | 222 | 225 | 1237.9 |
| (*A*a) | LMA | 2 | 223 | 225 | 1075.7 |
| | Area-based P(Pa) | 2 | 223 | 225 | 1048.7 |
| | Area-based N (Na) | 2 | 223 | 225 | 1051.6 |
| | Site, LMA | 4 | 221 | 225 | 1059.5 |
| | Site, Pa | 4 | 221 | 225 | 1027.7 |
| | Site, Na | 4 | 221 | 225 | 1029.1 |
| | LMA, Pa | 3 | 222 | 225 | 1046.7 |
| | LMA, Na | 3 | 222 | 225 | 1048.2 |
| | Pa, Na | 3 | 222 | 225 | 1036.8 |
| | Site, LMA, Pa | 5 | 220 | 225 | 1024.5 |
| | Site, LMA, Na | 5 | 220 | 225 | 1026.1 |
| | Site, Pa, Na | 5 | 220 | 225 | 1013.8 |
| | LMA, Pa, Na | 4 | 221 | 225 | 1029.3 |
| | Site, LMA, Pa, Na | 6 | 219 | 225 | 1005.2 |
| | Site, LMA, Pa, Na, Pa:Na | 8 | 217 | 225 | 1007.2 |
| | Site, LMA, Pa, Na, Site:Pa | 9 | 216 | 225 | 997.7 |
| | Site, LMA, Pa, Na, Site:Na | 9 | 216 | 225 | 1006.8 |
| Mass-based *A*sat | Site | 3 | 222 | 225 | 2177.6 |
| (*A*m) | LMA | 2 | 223 | 225 | 2050.2 |
| | Mass-based P (Pm) | 2 | 223 | 225 | 2068.3 |
| | Mass-based N (Nm) | 2 | 223 | 225 | 2087.4 |
| | Site, LMA | 4 | 221 | 225 | 2048.1 |
| | Site, Pm | 4 | 221 | 225 | 2058.4 |
| | Site, Nm | 4 | 221 | 225 | 2063.5 |
| | LMA, Pm | 3 | 222 | 225 | 2017.3 |
| | LMA, Nm | 3 | 222 | 225 | 2019.3 |
| | Pm, Nm | 3 | 222 | 225 | 2028.1 |
| | Site, LMA, Pm | 5 | 220 | 225 | 2007.4 |
| | Site, LMA, Nm | 5 | 220 | 225 | 2010.0 |
| | Site, Pm, Nm | 5 | 220 | 225 | 2012.5 |
| | LMA, Pm, Nm | 4 | 221 | 225 | 1996.0 |
| | Site, LMA, Pm, Nm | 6 | 219 | 225 | 1984.1 |
| | Site, LMA, Pm, Nm, Pm:Nm | 8 | 217 | 225 | 1975.1 |
| | Site, LMA, Pm, Nm, Site:Pm | 9 | 216 | 225 | 1973.5 |
| | Site, LMA, Pm, Nm, Site:Nm | 9 | 216 | 225 | 1984.0 |
